# Sex-specific hippocampus volume changes in obstructive sleep apnea

**DOI:** 10.1016/j.nicl.2018.07.027

**Published:** 2018-07-27

**Authors:** Paul M. Macey, Janani P. Prasad, Jennifer A. Ogren, Ammar S. Moiyadi, Ravi S. Aysola, Rajesh Kumar, Frisca L. Yan-Go, Mary A. Woo, M. Albert Thomas, Ronald M. Harper

**Affiliations:** a UCLA School of Nursing, University of California at Los Angeles, Los Angeles, CA 90095, United States; b Brain Research Institute, David Geffen School of Medicine at UCLA, University of California at Los Angeles, Los Angeles, CA 90095, United States; c Department of Neurobiology, David Geffen School of Medicine at UCLA, University of California at Los Angeles, Los Angeles, CA 90095, United States; d Medicine–Division of Pulmonary and Critical Care, David Geffen School of Medicine at UCLA, University of California at Los Angeles, Los Angeles, CA 90095, United States; e Anesthesiology, David Geffen School of Medicine at UCLA, University of California at Los Angeles, Los Angeles, CA 90095, United States; f Radiological Sciences, David Geffen School of Medicine at UCLA, University of California at Los Angeles, Los Angeles, CA 90095, United States; g Department of Neurology, David Geffen School of Medicine at UCLA, University of California at Los Angeles, Los Angeles, CA 90095, United States

**Keywords:** AHI, apnea-hypopnea index, CA, cornu ammonis, OSA, obstructive sleep apnea, Autonomic, Oxidative stress, Inflammation, Intermittent hypoxia, Neuroimaging

## Abstract

**Introduction:**

Obstructive sleep apnea (OSA) patients show hippocampal-related autonomic and neurological symptoms, including impaired memory and depression, which differ by sex, and are mediated in distinct hippocampal subfields. Determining sites and extent of hippocampal sub-regional injury in OSA could reveal localized structural damage linked with OSA symptoms.

**Methods:**

High-resolution T1-weighted images were collected from 66 newly-diagnosed, untreated OSA (mean age ± SD: 46.3 ± 8.8 years; mean AHI ± SD: 34.1 ± 21.5 events/h;50 male) and 59 healthy age-matched control (46.8 ± 9.0 years;38 male) participants. We added age-matched controls with T1-weighted scans from two datasets (IXI, OASIS-MRI), for 979 controls total (426 male/46.5 ± 9.9 years). We segmented the hippocampus and analyzed surface structure with “FSL FIRST” software, scaling volumes for brain size, and evaluated group differences with ANCOVA (covariates: total-intracranial-volume, sex; P < .05, corrected).

**Results:**

In OSA relative to controls, the hippocampus showed small areas larger volume bilaterally in CA1 (surface displacement ≤0.56 mm), subiculum, and uncus, and smaller volume in right posterior CA3/dentate (≥ − 0.23 mm). OSA vs. control males showed higher bilateral volume (≤0.61 mm) throughout CA1 and subiculum, extending to head and tail, with greater right-sided increases; lower bilateral volumes (≥ − 0.45 mm) appeared in mid- and posterior-CA3/dentate. OSA vs control females showed only right-sided effects, with increased CA1 and subiculum/uncus volumes (≤0.67 mm), and decreased posterior CA3/dentate volumes (≥ − 0.52 mm). Unlike males, OSA females showed volume decreases in the right hippocampus head and tail.

**Conclusions:**

The hippocampus shows lateralized and sex-specific, OSA-related regional volume differences, which may contribute to sex-related expression of symptoms in the sleep disorder. Volume increases suggest inflammation and glial activation, whereas volume decreases suggest long-lasting neuronal injury; both processes may contribute to dysfunction in OSA.

## Highlights

• The hippocampus in OSA shows areas of increased and decreased volume.
• The injury is sex-specific, in subregions related to symptoms in females and males.
• Injury may be inflammation (volume increases) or cell death (volume decreases).

## 1 Introduction

The hippocampus shows both damage and dysfunction in obstructive sleep apnea (OSA), which may contribute to memory, autonomic and depressive symptoms in the disorder. Early findings indicate volume reductions and other structural changes in or adjacent to the hippocampus (Macey et al., 2002; Dusak et al., 2013; Morrell et al., 2003; Tummala et al., 2017; Tummala et al., 2016), and metabolite levels suggestive of inflammation and glial activation (O'Donoghue et al., 2012; Sarma et al., 2014; Alkan et al., 2013; Kizilgoz et al., 2013; Algin et al., 2012; Bartlett et al., 2004). Patterns of activity within the structure are modified in OSA, as measured by functional neuroimaging (Henderson et al., 2003; Harper et al., 2003; Macey et al., 2003; Macey et al., 2006; Castronovo et al., 2009; Fatouleh et al., 2014; Li et al., 2016a; Li et al., 2016b), and are reflected in symptoms such as elevated sympathetic tone, high levels of depressive and anxiety symptoms, and memory difficulties (Narkiewicz and Somers, 2003; Hoth et al., 2013; Rezaeitalab et al., 2014). The particular areas of injury in the hippocampus are not well defined, nor are patterns of volume increase or decrease understood; inflammation and glial activation should produce volume increases, whereas neuronal death or damage to cells should cause volume decreases. Moreover, the symptoms expressed in OSA differ by sex, and it is unclear whether structural changes differ between males and females in a way that reflects those characteristics. Finally, hippocampal roles in some functions, such as regulation of blood pressure, are highly lateralized, and it is unclear whether OSA damage is equally expressed in both left and right hippocampi.

Knowing the location and nature of hippocampal volume changes would provide insights into mechanisms of pathology which accompany OSA. The multitude of potentially damaging processes occurring in OSA makes predicting volume changes in the disorder difficult, since neuronal death results in volume loss, but glial responses to hypoxia can increase tissue volume. Both of these effects are visible in the putamen in OSA (Kumar et al., 2014a), and similar patterns may occur in the hippocampus. Precise volumetric assessment is possible, but sensitivity is limited by small subject samples. However, a combination of publically-available MRI databases and analytic software now allow hundreds of subjects to be used as a population reference. Early hippocampal analyses required manual, time-consuming tracing of structures (Morrell et al., 2003; Macey et al., 2009; Thompson et al., 2004). Automatic segmentation methods include FreeSurfer (Fischl et al., 2002), its subsequent improvements (Clerx et al., 2015; Iglesias et al., 2015), and FSL FIRST (Patenaude et al., 2011), and these approaches allow analysis of large numbers of subjects in an objective, repeatable manner. In particular, FSL FIRST allows for shape analysis with group and regression analyses, and using conventional anatomical MRI scans can distinguish between hippocampal subregions of volume change measured as surface displacement at sub millimeter resolution.

Our objective was to assess OSA-related differences in regional hippocampal volume, relative to a large reference population, and to assess laterality, regional site, and sex-specific effects of OSA on hippocampal structure. Based on symptoms found in OSA patients, we hypothesized volume changes would appear in memory-, autonomic-, and mood-related subfields of the hippocampus. However, we did not hypothesize a direction of change, since available evidence suggests the potential for both acute (volume increases from inflammatory and other processes) and long-term (volume decreases and cell death) injury.

## 2 Methods

### 2.1 Subjects

We performed high-resolution T1-weighted imaging in 66 newly-diagnosed, untreated OSA (mean age ± SD: 46.3 ± 8.8 years; mean AHI ± SD:34.1 ± 21.5 events/h; 50 male) and 59 healthy age-matched control (46.8 ± 9.0 years; 38 male) participants. Further sleep and demographic details are in Table 1, Table 2. Sleep scoring was per the 1999 AASM criteria (Author, 1999). The study was approved by the UCLA Institutional Review Board, and all subjects provided written informed consent. To improve sensitivity, we combined our healthy control sample with two large datasets, IXI (https://www.nitrc.org/projects/ixi_dataset/) and OASIS (http://www.oasis-brains.org/; Marcus et al., 2007), resulting in a control group of 979 age-matched subjects (426 male, 46.5 ± 9.9 years) representative of the general population. These studies were approved by their governing ethics committees, and all subjects provided written informed consent.

**Table 1:** Characteristics of OSA and control subjects. Group differences assessed with independent samples *t*-tests for continuous variables, and chi-square for sex. Gray cells indicate not applicable.

	Mixed			Female			Male		
	OSA N = 65	Control population N = 980	OSA vs Control	OSA N = 15	Control population N = 553	OSA vs Control	OSA N = 50	Control population N = 426	OSA vs Control
		Mean ± std	P		Mean ± std	P		Mean ± std	P
Age (years)	47.5 ± 9.9	47.5 ± 18.8	0.6	51.4 ± 10.2	49.6 ± 19.2	0.7	44.9 ± 9.4	44.9 ± 17.7	1.0
Sex	16 ♀, 50♂	553 ♀, 426♂	<0.001

**Table 2:** Polysomonographic characteristics of OSA patients.

	Mixed	Female	Male
	OSA N = 65	OSA N = 15	OSA N = 50
	Mean ± std	Mean ± std	Mean ± std
AHI events/hour	30.6 ± 20.7	23.0 ± 22.4	33.0 ± 19.5
SaO2 (minimum %)	81.9 ± 9.1	86.9 ± 6.0	80.2 ± 9.4
SaO2 (baseline %)	94.7 ± 2.3	94.7 ± 2.4	94.7 ± 2.4

### 2.2 Implications of using a large reference dataset

While including the IXI and OASIS subjects means the control group will likely include some people with OSA, the large number of subjects leads to improved sensitivity to detect OSA-related effects. For example, a sensitivity analysis for ANCOVA shows that 125 subjects with a two group, three covariate model at alpha = 0.05 and power of 0.95 is sensitive to an effect size f of 0.46, whereas the same model with 1045 subjects is sensitive to an effect size f of 0.15. The main consequence of the undetected OSA in the control group would be to reduce the OSA-control group differences, and hence underestimate the magnitude of any effect. A further advantage of using these datasets as a population reference is that other researchers can compare their findings against a common standard.

### 2.3 MRI protocol

Image volumes for the UCLA subjects were acquired on a Siemens 3 Tesla Trio scanner with magnetization prepared rapid acquisition gradient echo protocol product sequence (MPRAGE; TR = 2200 ms, TE = 2.34 ms, inversion time = 900 ms, flip angle = 9°), with 320 × 320 matrix size, 230 × 230 mm field of view (FOV), 0.9 mm slice thickness, 192 sagittal slices, and two repeats. An acceleration factor of two was applied with generalized-autocalibrating-partially-parallel-acquisition parallel imaging (GRAPPA).

### 2.4 Analysis

All T1-weighted image scans were visually inspected for artifact and signs of movement. Scans were manually rigid-body aligned to the MNI template using the SPM “Display” function, with rotations and x/y/z shifts. FSL FIRST processing was then applied, which segments the hippocampus and assesses regional surface structure (Patenaude et al., 2011). Visual assessment of each scan's registration and segmentation accuracy was performed. In cases where subjects did not have accurate brainstem segmentation, they were nevertheless retained if the hippocampus was accurately segmented. The FIRST algorithm produces similar segmentations as manual outlining (Mulder et al., 2014; Perlaki et al., 2017), with the advantage of being reproducible independent of expert effort. The FIRST method also provides reproducible results (Bartel et al., 2017). That said, our experience is that the anatomical scans need to be rigid-body realigned to template space, as otherwise, segmentation with FIRST (and other algorithms) can fail.

Hippocampal volumes were scaled for total brain size based on registration to a common space (6 parameter affine normalization), and included total intracranial volume (TIV) and sex as covariates (ANOVA; P < .05, false discovery rate [FDR] correction for multiple comparisons). TIV was derived from the SPM segmentation of gray and white mater and cerebrospinal fluid, with voxels classified based on the sum of probabilities of the three tissue types ≥0.5.

**Fig. 1:**
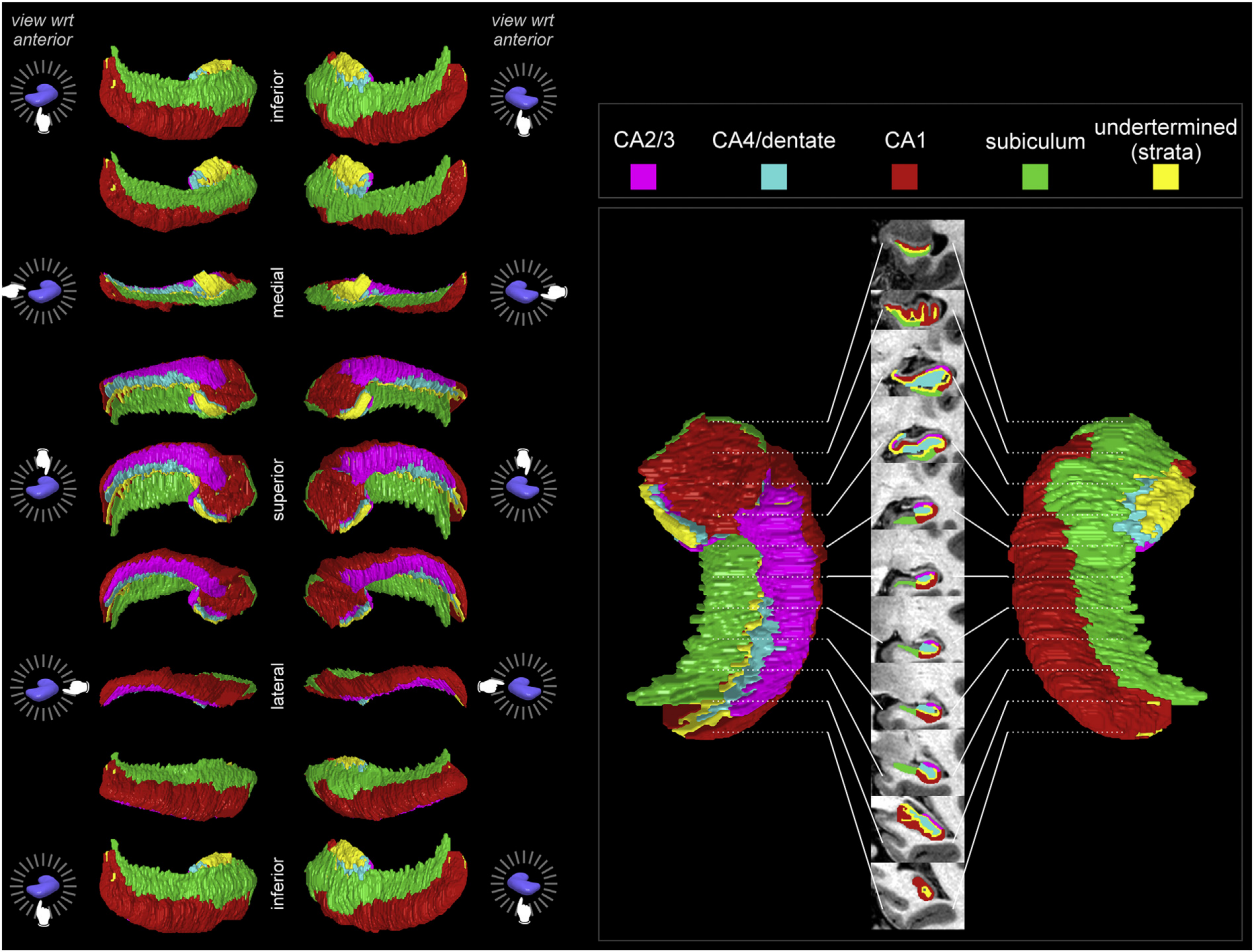
Hippocampal subfields based on the Winterburn atlas [subject 1; 37]. Left panel shows 45° rotations around anterior-posterior axis for surfaces of left and right hippocampi. Right panel shows right hippocampus dorsal (left) and ventral (right) representations, and the center shows coronal slices along anterior-posterior axis with the subregions overlaid onto the subject's high-resolution T1-weighted anatomical scan included with the Winderburn atlas.

**Fig. 2:**
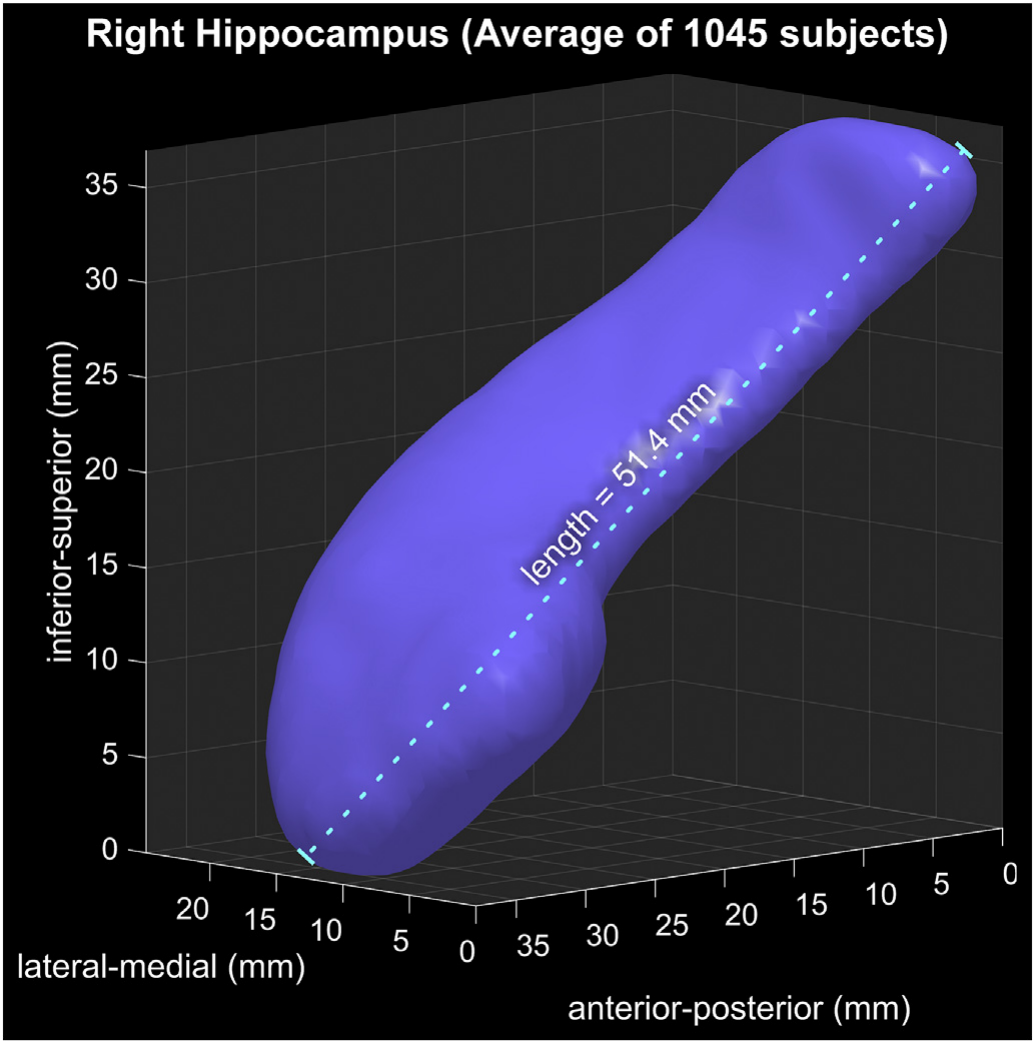
Reconstruction of average of 1045 right hippocampal segmentations, illustrating shape and size of the “template” hippocampus against which individual surface displacements are measured.

**Fig. 3:**
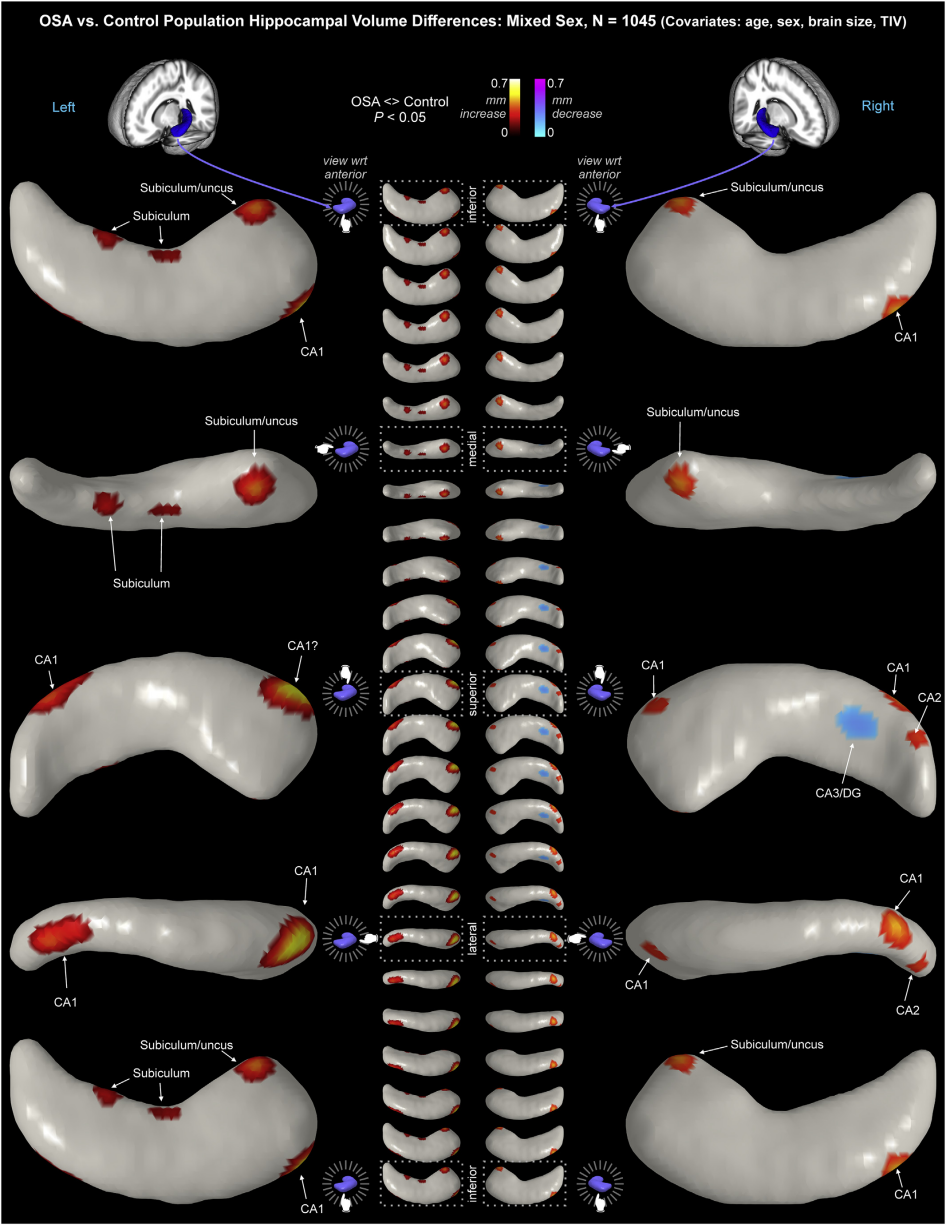
OSA hippocampal volume changes relative to controls in a mixed group (males and females). Regions of significant displacement (P < .05, corrected for multiple comparisons) are color-coded according to average displacement. Warm colors reflecting outward displacement, i.e., volume increases, and cool colors reflect inward displacement, i.e., volume decreases (key at top). The model includes age, sex and TIV as covariates. The left hippocampus is on the left side of the figure, and the right hippocampus on the right of the figure. The panel shows views with the structure rotation about the anterior-posterior axis. The large images are 90° rotations and the smaller middle images are 15° rotations.

Surface regions showing significant deviations were visualized with MRIcroS (https://www.nitrc.org/projects/mricros/), a MATLAB-based surface rendering package developed from concepts in MRIcroN software (Rorden et al., 2007). We modified the MRIcroS code to allow display of volume increases as warm colors and decreases as cool colors. We also enabled scaling of the overlays, such that the effect size in millimeter deviation could be shown. To localize the affected subfields, we used a standard atlas (Mai et al., 2004) to infer the dominant areas underlying any particular surface point. We also created a visual guide using a freely-available high-resolution hippocampal atlas (Winterburn et al., 2013). The “Winterburn” atlas consists of very high-resolution T1 and T2 scans (0.3 mm^3^ voxel size), with hippocampal subfield segmentations (CA1, CA2/3, CA4/dentate, undetermined/strata, and subiculum; CA = Cornu ammonis). Using MRIcroS, we created volume renderings of the subfields (Fig. 1), which illustrate the principal subfields underlying each surface location. For example, the inferior surface reflects the subiculum along the medial aspect and CA1 along the lateral aspect [see also Fig. 2 in (Ogren et al., 2009). Link: http://www.ncbi.nlm.nih.gov/pmc/articles/PMC2773143/figure/F2/]. However, changes in some surface regions likely reflect the volume of deeper subfields, as shown by the cross sections in Fig. 3. We used the Mai atlas (Mai et al., 2004) to infer regions most likely represented at surface locations, based on the relative proportion of each subregion under the hippocampal surface.

### 2.5 Sex differences

To provide context for the sex-specific effects, we also described the sex differences within the control and OSA groups. For each group, the female vs male effects were calculated and presented using the same analysis approach.

### 2.6 Platform influences

All hippocampi segmentations were manually verified. However, to further address possible influences of scanner and platform variations, we calculated descriptive statistics (mean and standard deviation) for age, TIV, and brain volume by platform. We separated our UCLA scans into two “platforms” since a scanner upgrade was performed part way through the study, although evidence suggests such upgrades have little effect on variability (Jovicich et al., 2009). Brain volume was calculated as voxels where the combined probability of gray and white matter was ≥0.5. We did not include platform as a covariate since this action would reduce sensitivity (Takao et al., 2014), and these measures were intended as a description, rather than a correction of possible scanning influences.

## 3 Results

### 3.1 Quality control

Quality control revealed hippocampal segmentation problems in one IXI and four OASIS subjects (numbers IXI #600, OASIS #80, #147, #270, #370), which were excluded from analysis. The remainder were deemed to have accurate segmentations of the structure. Fig. 2 illustrates the reconstructed average surface of the right hippocampus from the 1045 mixed (i.e., all male and female) subjects, which had a length of 51.4 mm (in common space). Other metrics of reconstructed sizes are shown in Table 3, illustrating slight variations in size between sexes and between left and right sides. The reported sizes reflect brain scans normalized to MNI space via FLS FIRST processing. Thus, the larger female values for left and right hippocampi reflect structures that are larger proportionally (relative to overall brain size), as opposed to native, unscaled sizes where female values would likely be smaller, due to known smaller overall brain volumes relative to males (Leonard et al., 2008; Luders et al., 2009).

**Table 3:** Sizes of mean MNI-space hippocampi calculated by FIRST.

Size of mean hippocampi	Left		Right	
	Surface voxels 1 mm^3^	Length mm	Surface voxels 1 mm^3^	Length mm
Mixed	6498	51.39	6480	51.44
Female	6597	51.46	6567	51.44
Male	6378	51.20	6356	51.44
Difference (male - female)	−219	−0.263	−211	0

### 3.2 Platform variations

**Supplementary Fig. 1:**
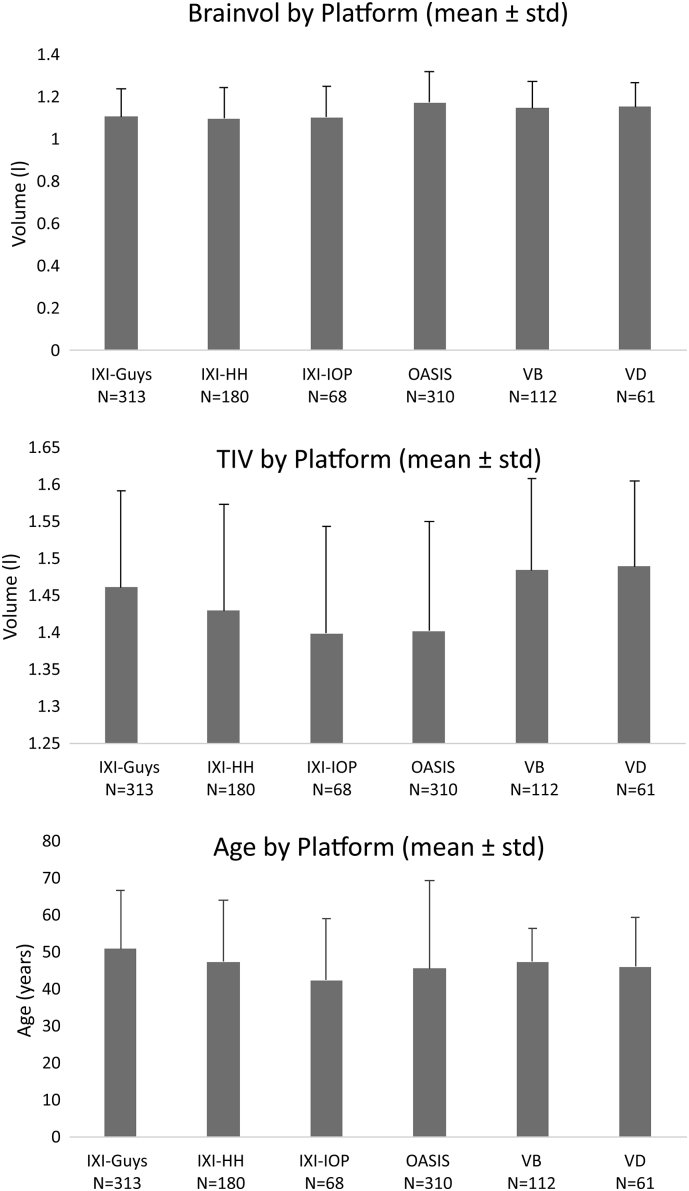
Platform variations in measures of brain volume (Brainvol), total intracranial volume (TIV), and age.

There were six groups separated by platform: the three IXI scanning sites, the OASIS set, and the UCLA scans collected under both the “VB” and “VD” platforms. Supplementary Fig. 1 illustrates the mean and standard deviation for age, TIV and brain volume. Overall values were similar by platform. TIV varied most substantially, likely reflecting the variation in scanning volume, with scanning protocols that extend down the neck probably leading to more CSF voxels being included as TIV. However, the brain volume values, arguably the most important indicator of systematic variation, were very similar across platforms.

### 3.3 Mixed-sex OSA findings

Significant overall changes in the mixed OSA group appeared in a number of hippocampal regions (P < .05, FDR control for multiple comparisons), controlling for age, sex, and TIV. Obstructive sleep apnea was accompanied principally by volume increases, reflected as surface displacement from the mean up to 0.56 mm in subfields of the left and right hippocampi, and some regions of volume decrease, reflected as surface displacement from the mean to −0.23 mm (Table 4; Fig. 3). The regions showing volume increases included the bilateral anterior subiculum extending to the uncus, with more substantial increases on the left side (Table 4; Fig. 3). The left side also showed two small regions of volume increase in the mid-hippocampal area of the subiculum on the medial aspect. The posterior CA1 in the lateral region was affected bilaterally, again with greater changes on the left side. Changes specific to the right hippocampus included a small area of volume increase in CA2 of the posterior hippocampus, close to the tail. A volume decrease was present in CA3/dentate of the posterior hippocampus.

**Table 4:** Effect size ranges for significant OSA-control differences. Units are mm surface displacement from mean. ns = not significant.

Effect size (mm)		Increase		Decrease	
		Min	Max	Min	Max
Mixed	Left	0.161	0.559	ns	ns
	Right	0.285	0.455	0.156	0.231
Female	Left	ns	ns	ns	ns
	Right	0.059	0.674	0.080	0.518
Male	Left	0.042	0.582	0.043	0.204
	Right	0.039	0.609	0.045	0.233

### 3.4 Female OSA findings

**Fig. 4:**
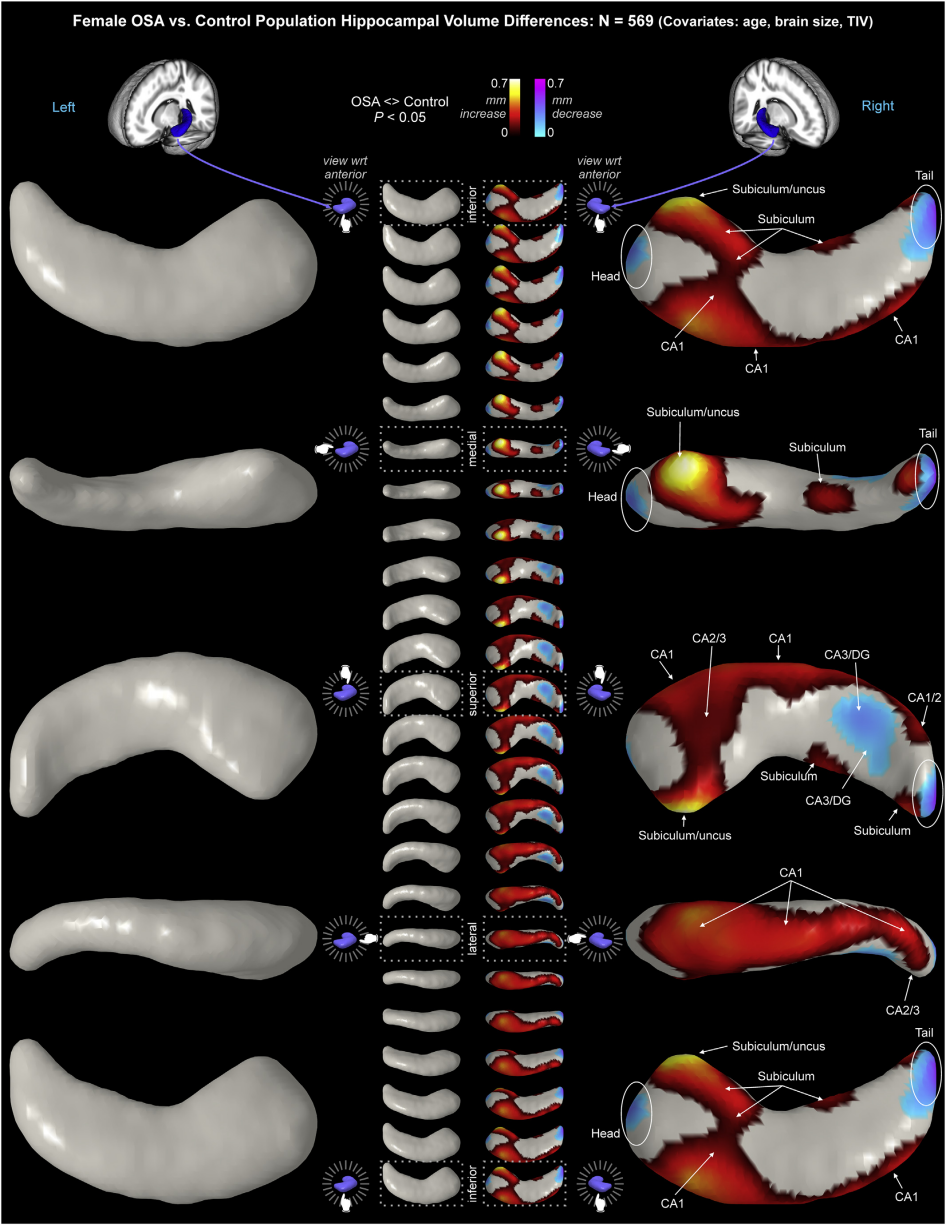
Female OSA hippocampal volume changes relative to controls, with age and TIV as covariates. Figure conventions as in Fig. 3.

Female OSA subjects showed right-sided volume declines, with up to −0.5 mm surface reductions, and volume increases up to 0.67 mm (Table 4; Fig. 4). Sex-specific OSA-related changes were substantially more extensive than in the mixed group, with larger maximum effect sizes (Table 4). However, no changes were significant in female OSA patients in the left hippocampus after FDR correction for multiple comparisons (Table 4; Fig. 4). On the right side, extensive areas of volume increase appeared, with the largest in the anterior subiculum and uncus. Increased volumes also appeared in the medial subiculum in the mid-hippocampal regions, and a small area near the tail. The entire lateral aspect of CA1 showed increased volume from anterior to posterior. CA2/3 in the anterior hippocampus showed increased volume. Volume declines appeared in the head and the tail, and in CA3/dentate in the posterior region.

### 3.5 Male OSA findings

**Fig. 5:**
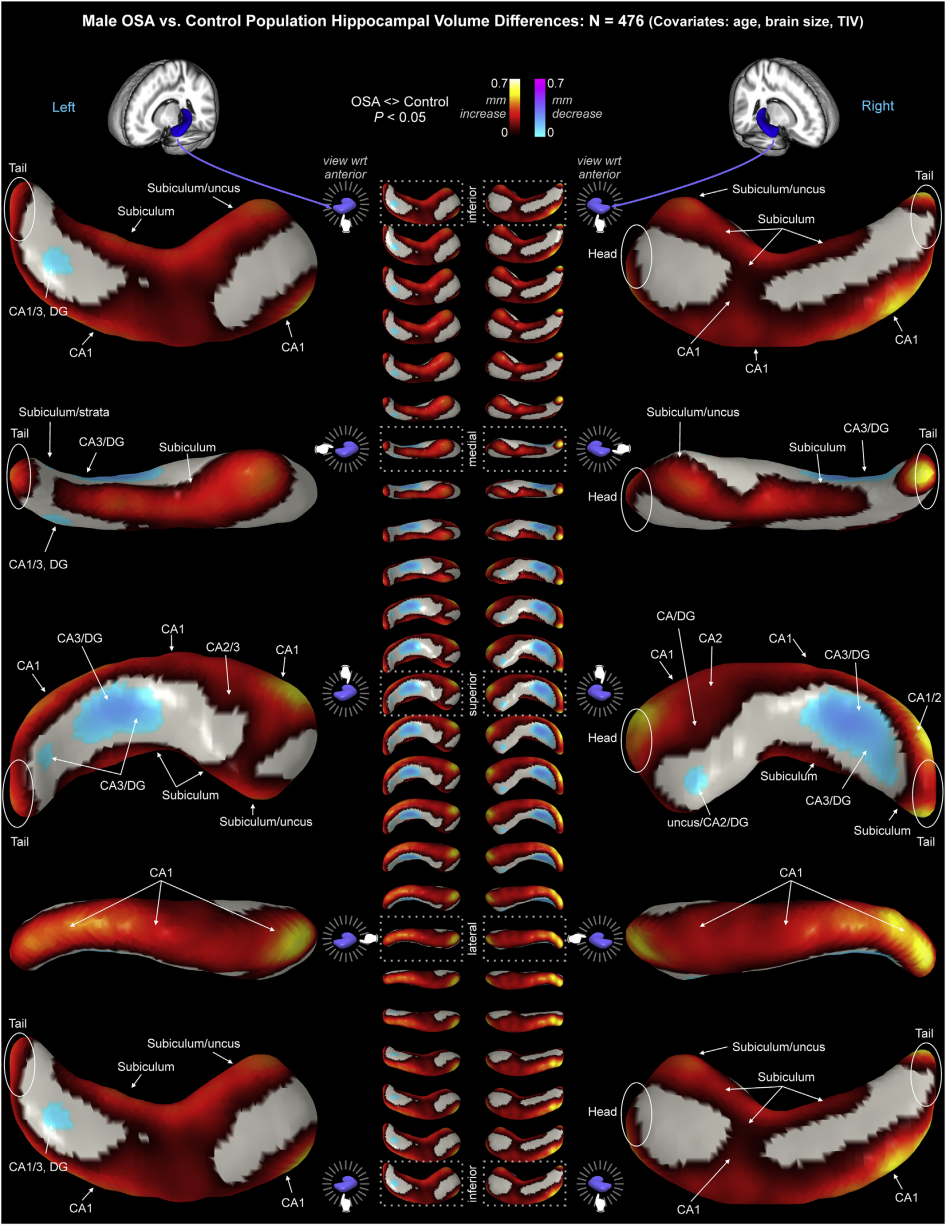
Male OSA hippocampal volume changes relative to controls, with age and TIV as covariates. Figure conventions as in Fig. 3.

Male OSA showed left-sided volume decreases up to −0.20 mm, and volume increases up to 0.58 mm (Table 4; Fig. 5). On the right side, volume declines up to −0.23 mm and volume increases up to 0.61 mm appeared. The male OSA bilateral volume variations had similar distributions on both sides. Volume increases appeared through CA1, extending to the head and tail regions, with the magnitude of increases larger on the right. The subiculum was larger from mid-to-anterior hippocampus, extending to the uncus in the anterior portion. Anterior CA2/3 showed volume increases. Bilateral volume decreases appeared in CA3/dentate in the posterior hippocampus, with changes visible on the superior surface bilaterally, and also on the inferior surface on the left side.

### 3.6 Model fit and residuals

**Fig. 6:**
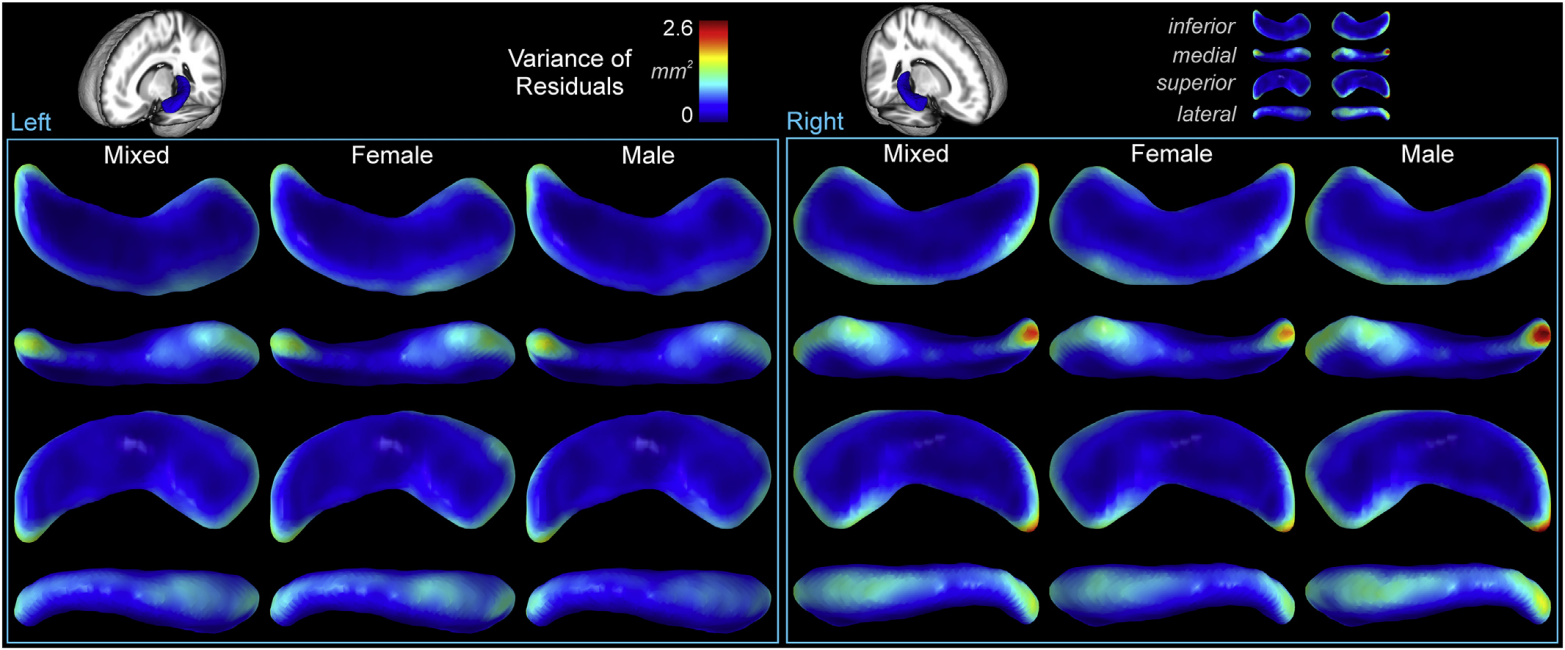
Variance of residuals in mm^2^, illustrating higher variability in the tail, moderate variability in the head, and low variability in the body. The right side showed higher variability than the left, and males showed higher variability than females.

Lower variance of residuals indicates better model fit. The variance of the residuals was greatest in the tail of the hippocampus (Fig. 6). The medial head of the hippocampus also showed higher residual variability than other areas, although the magnitude was approximately half that of the tail (right side: ~1.2 mm^2^ versus ~2.6 mm^2^). The inferior and superior surfaces showed very low residual variance, and medial and lateral aspects showed moderate levels. The right side residual variance was substantially higher than the left. Females showed slightly lower variance than males (Table 5).

**Table 5:** Variance of residuals.

Variance mm^2^	Sum over voxels		Variance/voxel		Max voxel variance	
	Left	Right	Left	Right	Left	Right
Mixed	1119	1187	0.172	0.183	1.49	2.19
Female	1071	1085	0.162	0.165	1.42	1.95
Male	1167	1288	0.183	0.203	1.63	2.63

### 3.7 Sex differences in control and OSA groups

**Fig. 7:**
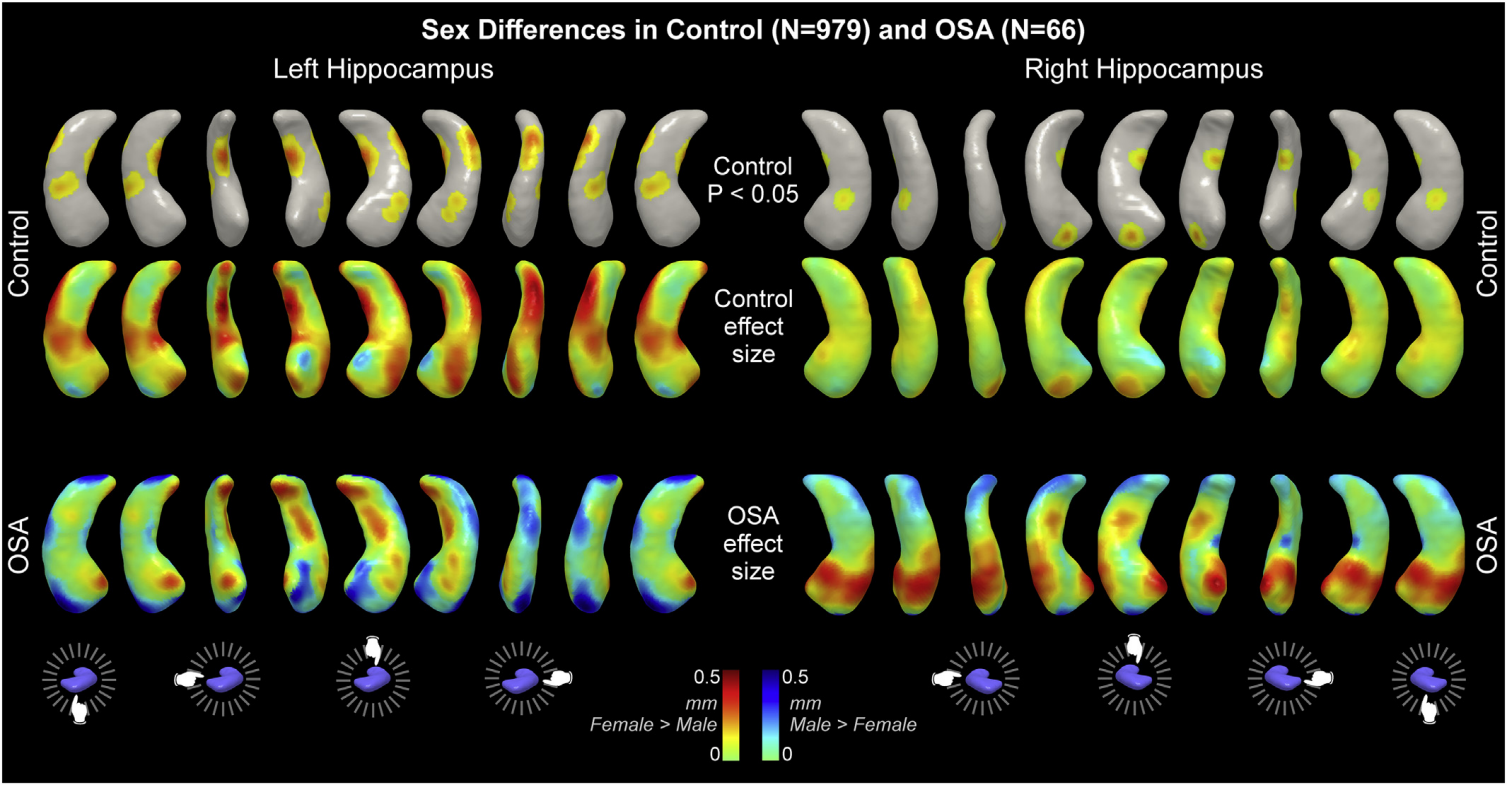
Sex differences in control and OSA groups. Areas of significantly higher volume in female vs male in controls are shown in top row (P < .05). Effect sizes for all differences are shown for control (middle row) and OSA (bottom row). See Table 1 for subject details.

We assessed female and male differences separately within control and OSA groups. Table 1 shows the group numbers and breakdown for female and male subjects. The OSA group did not show significant effects, likely because the sensitivity was relatively low due to the small sample size (Table 6). The control group showed regions of significantly high volume in female vs males (Table 6 and Fig. 7). Since the model includes scaling by head size and TIV as a covariate, these findings represent relative variations, as opposed to absolute differences in volume. The effect sizes in OSA were larger than control (Table 6), and the patterns of male-female difference were distinct in OSA and control groups (Fig. 7). Of note, multiple regions of higher relative volume in females in the control group (darker red in middle row of Fig. 7) showed opposite effects in the OSA group (blue in bottom row of Fig. 7).

**Table 6:** Effect size of sex differences in control and OSA groups, based on comparisons with TIV as covariate and whole-brain scaling. Units are mm surface displacement from mean. Effects with Min/Max are significant (p < .05, corrected). ns = not significant; effect size of maximum shown.

Effect size (mm)		Female < Male		Female > Male	
		Min	Max	Min	Max
OSA N = 66	Left	ns max effect size = 0.51		ns max effect size = 0.36	
	Right	ns max effect size = 0.36		ns max effect size = 0.45	
Control N = 979	Left	ns max effect size = 0.0003		0.123	0.291
	Right	ns max effect size = 0.0001		0.088	0.216

## 4 Discussion

### 4.1 Overview

Obstructive sleep apnea is accompanied by isolated areas of bilateral hippocampal volume increases in CA1, subiculum and uncus, and volume decreases in right CA3/dentate in the posterior hippocampus. The alterations show extensive sex-specific changes. Males show bilateral volume increases through the extent of CA1 and subiculum, with greater changes on the right side. The volume increases extend to the head and tail of the structure. Bilateral volume decreases in CA3/dentate appear in the mid and posterior hippocampus. Females show significant effects only on the right side, with CA1 and subiculum/uncus volume increases and posterior CA3/dentate volume decreases, as in males. However, in contrast to males, females with OSA show volume decreases in the right head and tail of the hippocampus. The findings demonstrate the importance of considering sex in the assessment of OSA-related brain changes, since mixed groups masked the extent of volume alterations linked to the sleep disorder. Additionally, the different patterns of change in females and males may be reflected in the differing symptoms between the sexes. Of particular importance is that certain functions, especially those related to autonomic regulation, are typically lateralized, and the asymmetric damage found in females may contribute significantly to sex-based differences in symptoms in OSA.

### 4.2 Discussion of findings

The sex-related differences in volume change patterns suggest that separate neuropathologies contribute to symptom characteristics in females and males with OSA. Another possibility is that OSA is diagnosed later in females, and hence more chronic injury may be present in that group. However, this possibility would not account for the site-specific nature of the volume reductions. Depression is elevated in OSA, but more so in females vs. males (Macey et al., 2010), and the hippocampus plays significant roles in depressive disorders and symptom levels in the general population (Neumeister et al., 2005; Bremner et al., 2000). Depression in females is linked with greater morphological changes in the right over the left hippocampus (Tae et al., 2011). Since the primary volumetric changes in OSA females appeared on the right side, lateralization of tissue change may contribute in some fashion to initiating or maintaining depression signs in OSA females. The dentate shows volume reductions in the posterior region, and in the right head and tail in female OSA subjects. The findings suggest cell loss in these regions, which presumably impact neural functions performed by the structure. The pathophysiology may also differ by sex: astrocytic swelling following hypoxia is attenuated by estradiol (Rutkowsky et al., 2011), which is approximately four times higher in females than males (Elmlinger et al., 2002). Estradiol and other sex hormones, which influence both pathological and normal processes (McCarthy et al., 2003), likely underlie at least some of the gender-specific volume changes.

The sex differences within the control group show that females and males have different baseline hippocampus sizes, so when combined with likely sex-specific OSA influences, there is little understanding to be gained from OSA female vs. male comparisons.

Decreased volume occurs in the presence of cellular loss or shrinkage, which in adults is usually associated with age-related atrophy or neurodegenerative processes, with the hippocampus being especially affected (Hof and Morrison, 1996; Raz et al., 2005). Excitotoxicity, common in epilepsy, is a suspect mechanism, given the excitotoxic mechanism triggered by hypoxia (Fung et al., 2012; Fung et al., 2007), and the high levels of insular glutamate in OSA (Macey et al., 2016). However, in epilepsy, excitotoxicity leads to decreased volumes in CA1 (Kim et al., 2015), and thus does not explain the volume increases in that subfield found here. Increased volumes in any brain region can arise from inflammation in the acute phase (Cheriyan et al., 2012), a process likely operating in OSA (Chen et al., 2015); over the long term, inflammation is accompanied by volume decreases (Braskie et al., 2014; Jefferson et al., 2007). Animal models show hippocampal inflammation in response to intermittent hypoxia (Sapin et al., 2015). Astrocyte activation is also likely present in OSA, since animal models show glial swelling in response to intermittent hypoxia (Rumpel et al., 1997; Kempski and Volk, 1994). Such volume increases could occur in white or gray matter, given the distribution of glia in both tissue types. Gray matter-specific changes can also arise from dendritic growth through “learning” or other functional requirements; that aspect is suggested by the findings of enlarged hippocampi in London taxi drivers, attributed to the spatial learning needed for that profession (Maguire et al., 2000). Neurogenesis is another potential source of volume increase, with such processes especially prominent in the hippocampus (Eriksson et al., 1998). Based on animal models and patient symptoms, inflammation and astrocyte activation are the most likely sources of OSA volume increases reported here, whereas cellular loss and shrinkage, especially in neurons, are the most likely sources of volume decreases.

Previous assessments of the brain in OSA showed varying hippocampal findings, including both left (Morrell et al., 2003) and right (Macey et al., 2002) volume reductions, the latter together with bilateral parahippocampal gyri volume reduction. Similar tissue loss was found by other groups (Yaouhi et al., 2009). Reduced gray matter was found in the bilateral anterior hippocampus, perhaps reflective of reduced cell density (Joo et al., 2010). Metabolite changes in the left hippocampus include higher N-Acetylaspartic acid (NAA)/creatine ratios (Alkan et al., 2013), possibly stemming from reduced creatine, which is associated with lower cell density. A pilot study showed lower NAA in the left hippocampus (Sarma et al., 2014), but the OSA group included treated subjects, and the location may have overlapped with frontal white matter, which several studies show to have lower NAA (Alchanatis et al., 2004; Sarchielli et al., 2008; Kamba et al., 2001; Kamba et al., 1997; Tonon et al., 2007). Diffusion tensor imaging indicates that females with OSA show reduced axonal integrity in the mid-hippocampal area (Macey et al., 2012). Mean water diffusivity has shown both lower (Kumar et al., 2012) and higher (Emin Akkoyunlu et al., 2013) values, perhaps from acute pathology (lower values) versus chronic neurodegeneration (higher values). More advanced diffusion assessment indicates myelin and axon damage in the hippocampus (Tummala et al., 2016; Kumar et al., 2014b). Cerebral blood flow findings are variable, with reductions in severe middle-aged OSA patients in the right hippocampus (Innes et al., 2015), no change in another sample of recently-diagnosed patients (Yadav et al., 2013), and increases in an older OSA group (Baril et al., 2015). All of these findings, however, combine male and female subjects for analyses, with little consideration of sex.

The hippocampal alterations presumably contribute to altered function related to OSA symptoms, especially impaired verbal memory, a common cognitive issue noted in OSA (Bucks et al., 2013; Hrubos-Strom et al., 2012). Hippocampal volume loss also appears in untreated depression (Bremner et al., 2000; Campbell et al., 2004), a finding which may be reversed with treatment (Sheline et al., 2003; Malykhin and Coupland, 2015). The hippocampus serves significant roles in autonomic, especially blood pressure, regulation and respiration (Cragg, 1958; Shoemaker et al., 2015; Ruit and Neafsey, 1988), both impaired in OSA (Macey et al., 2013; Narkiewicz et al., 1998). The rodent dorsal hippocampus, which corresponds to the hippocampal tail in humans (Sasaki et al., 2004), is particularly involved with autonomic regulation (Scopinho et al., 2013), and fMRI evidence shows impaired responses to respiratory, memory, and blood pressure challenges, as well as altered resting state (Henderson et al., 2003; Harper et al., 2003; Macey et al., 2003; Macey et al., 2006; Castronovo et al., 2009; Fatouleh et al., 2014; Li et al., 2016a; Shoemaker and Goswami, 2015).

The characteristics of how water diffuses through brain tissue in OSA provide evidence as to the nature of the hippocampal volume changes found here. Diffusivity, measured in an MRI scanner with diffusion tensor imaging procedures, is inversely related to intercellular barriers to water movement (Le Bihan et al., 2001). Lower mean diffusivity typically reflects increases in cell size or densities, such as occur with inflammation or astrocyte activation, whereas higher mean diffusivity reflects smaller or fewer cells, and is consistent with cell injury or death. Lower mean diffusivity (or the equivalent apparent diffusion coefficient) was found in some studies of OSA (Tummala et al., 2016; Emin Akkoyunlu et al., 2013; Kumar et al., 2014b), but others found no change (Algin et al., 2012), or an increase (Emin Akkoyunlu et al., 2013). One explanation for the divergent findings is that the populations with high diffusivity were at a later stage of the disorder, or had additional comorbidities, whereas the lower diffusivity occurred in OSA patients with a more recent development of the disorder. Consistent with this possibility, our study showing lower diffusivity (Kumar et al., 2012) had only recently-diagnosed OSA patients with no other chronic conditions. Diffusion measurements also highlight OSA-related alterations in structural connections to the hippocampus (Macey et al., 2008), including sex-specific differences (Macey et al., 2012).

Neurochemical levels measured with magnetic resonance spectroscopy are also sensitive to hippocampal changes in OSA, with alterations in metabolic state and structural composition consistent with inflammation and glial activation (O'Donoghue et al., 2012; Sarma et al., 2014; Alkan et al., 2013; Kizilgoz et al., 2013; Algin et al., 2012; Bartlett et al., 2004). The standard treatment, continuous positive airway pressure (CPAP), increases dentate volume after 8 or more months in some OSA populations (Kim et al., 2016), and reverses hippocampal metabolic changes potentially related to inflammation after 12 months (O'Donoghue et al., 2012). The evidence is therefore suggestive of acute structural changes in the hippocampus occurring early in OSA, which over time develop into cellular damage. This possibility suggests early CPAP or other interventions may minimize the development of hippocampal functional deficits.

Clinical implications of the findings include the possible contribution of OSA to cognitive decline and subsequent Alzheimer's disease (AD). OSA is associated with AD pathophysiology, including amyloid burden and atherosclerosis (Polsek et al., 2018; Lutsey et al., 2018; Sharma et al., 2018), and there is some evidence sleep-disordered breathing precipitates dementia (Lutsey et al., 2018; Osorio et al., 2015; Emamian et al., 2016). Additionally, brain changes in OSA impact structures that are associated with AD-related cognitive decline (Lutsey et al., 2016; Kerner et al., 2017), and the hippocampal changes shown here are another such finding. An intriguing possibility raised by the sex differences in OSA-related hippocampus volume changes is that the sleep disorder confers different risks for AD in females and males, a possibility raised over 30 years ago (Smallwood et al., 1983). The greater magnitude of volume declines in females shown here may relate to the greater impact of AD in females (Laws et al., 2016; Laws et al., 2018). Considering the subregions of the hippocampus affected in OSA, a meta-analysis of combined structural and functional alterations shows a region overlapping the medial CA1 (perhaps extending into the dentate) affected [Fig. 2A in (Tahmasian et al., 2016)], consistent with the present findings. The CA1, CA3 and dentate subfields have also been shown to be altered in AD (Yassa et al., 2010; Carmichael et al., 2012), so the combined findings are consistent with a neurobiological underpinning to the OSA as a risk factor for AD.

### 4.3 Limitations

The subjects in the IXI and OASIS datasets were not screened for OSA; thus, the control group probably included individuals with sleep-disordered breathing. The likely impact would be to reduce the sensitivity of the method to detect OSA-related changes, but the large number of control subjects should minimize the impact of undetected sleep disturbances (see Methods). A further possible confound of the population dataset is scanning variations. While such variations may be present, the brain volumes were consistent across platforms. Furthermore, the population dataset provide a common reference for other researchers. The identification of subfields based on the location of surface displacement is a simplification, since multiple subfields underlie each point on the surface. However, the location and magnitude of surface displacement measures are precise with this methodology (Patenaude et al., 2011).

### 4.4 Conclusions

The hippocampus shows sex-specific regional volume increases and, to a lesser extent, volume decreases in OSA, with increases largely in CA1 and decreases largely in dentate. The hippocampus in OSA shows increased bilateral volume in anterior and posterior lateral areas, and left medial mid-to-posterior sites, as well as volume decreases in right mid-to-posterior regions. Female volume changes were principally right-sided, an asymmetry that may contribute to the autonomic and depression differences relative to males. Volume increases suggest inflammation and glial activation, while declines could arise from localized neuronal injury. Sites of volume increase appeared in depression-related areas, whereas right-side volume decline sites mediate some cognitive processing functions. These hippocampal changes suggest at least some of the common symptoms in OSA, including sex-specific comorbidities, may be driven by damage-induced dysfunction in hippocampal subregions, and thus the structure is a potential target for neuroprotective interventions.

The following are the supplementary data related to this article.

## Funding sources

Supported by the National Institute of Nursing Research NR-013693. OASIS data collection supported by the P50 AG05681, P01 AG03991, R01 AG021910, P20 MH071616, and U24 RR021382.

## Declarations of interest

None.
